# Supplementary material for: The impact of visually simulated self-motion on predicting object motion
Source: PLoS One. 2024 Mar 14;19(3):e0295110. doi: 10.1371/journal.pone.0295110 (PMC10939277; doi:10.1371/journal.pone.0295110)
Supplement: Attachment — Submitted filename: Response to Reviewers.docx [file pone.0295110.s002.docx]

**Reviewer #1:**

*The authors may want to add some discussions on the design of the stimuli and explain how conclusions from studies tested on different stimuli may apply to this study. In this study, a lateral self-motion is simulated and the object motion is parallel to the observer motion. In other studies of flow parsing, a radial flow (e.g. Warren & Rushton, 2007; Dokka et al., 2015) or a rotational (e.g. Rushton & Warren, 2005) self-motion are often simulated. I am not sure if flow parsing for e.g. radial flow is the same as “flow parsing” for lateral flow because the situation seems is more complicated for a radial flow. It is possible in a simple situation like in this study, local motion contrast (Niehorster & Li, 2017) may play more role than flow parsing. Perception of object motion when the object motion is parallel to the self-motion might also be a special case because in real-life, we may never have to parse the flow if the object is in parallel as there is no danger of the object hitting us.*

RESPONSE: Lateral, rotational and forwards/backwards self-motion are indeed fairly different in terms of the geometry and in terms of their ecological usefulness. Nonetheless, findings are fairly consistent in that visually simulated self-motion alone usually leads to incomplete flow parsing and the corresponding biases in object motion judgements. We have added a section in the discussion to address these questions.

**Reviewer #2:**

*1) The main concern is that there is no real self-motion cues such as vestibular, efference copy signals are provided. It is not too surprising that visual alone would cause bias due to non-perfect flow parsing. Could these experiments be applied during VR when locomotion is accompanied?*

RESPONSE: The finding that visual input alone led to incomplete flow parsing is, as the reviewer notes rightly, unsurprising. We have shown this before, as well as a series of other studies. The main focus of this study was to investigate the link between incomplete flow parsing for a more perception-oriented task and a more action oriented (prediction task). Given our results, it would seem to us that the most interesting hook for future studies (with vestibular and/or active observer motion) lies in our observation of lower-than-expected variability in the prediction task for visual observer motion, which motivated us to posit a potential compensatory mechanism. It would be extremely interesting how this effect holds up when there are more self-motion cues available. (Dokka et al., for example, found that increased flow parsing leads to increased noise in object motion judgements; on the other hand, if there is indeed a compensatory mechanism, it might be stronger for more realistic situations.) We have added these thoughts in the conclusions section of the paper as possible future directions for this line of research.

*2) What about the effects when the visual flow is full of the display, instead of only on the ground?*

RESPONSE: It has been shown (Niehorster & Li, 2017) that flow parsing can decrease when there is no local information available to the observer. This is the case in our study, so we would expect flow parsing to be somewhat more complete if local optic flow information were also available to the observer.
